# Musculoskeletal disorders among preschool teachers: analyzing the relationships among relational demands, work meaning, and intention to leave the job

**DOI:** 10.1186/s12891-018-2081-z

**Published:** 2018-05-22

**Authors:** Daniela Converso, Sara Viotti, Ilaria Sottimano, Vincenza Cascio, Gloria Guidetti

**Affiliations:** 1 Department of Psychology, University of Turin, Via Giuseppe Verdi 8, 10124 Turin, Italy; 2 ASL TO1, Turin, Italy

**Keywords:** Musculoskeletal disorders, Relational demands, Preschool teachers, Conservation of resources theory

## Abstract

**Background:**

Based on the conservation of resource (COR) theory by Hobfoll, the aim of the present study was to test whether the relationships among relational demands, work meaning, and intention to leave vary as a function of the presence of musculoskeletal disorders (MSDs).

**Method:**

The study was cross-sectional and non-randomized. Analyses were carried out on a dataset consisting of 429 preschool teachers, who filled out a self-report questionnaire. Of them, 238 reported a MSD diagnosis and 191 were free form MSDs.

**Results:**

As expected, among those who reported MSDs, relational demands were significantly associated to intention to leave, and this relationship was mediated by work meaning; moreover, among those free from MSDs, no significant paths among the three variables were found.

**Conclusion:**

In general, results showed that suffering from MSDs impairs workers’ ability to face to relational demands, thus activating a spiral that encompasses diminished work meaning and intention to leave. Practical implications of results will be discussed in the paper.

## Background

Preschool services are essential in the modern societies since they have a strategic role, in concert with families, in sustaining children’s whole development (e.g., emotional, social, physical, and cognitive). In this context preschool teachers, who are professionals, mostly women, properly trained to fulfill these aims, carry out a job primarily of a relational nature, consisting of nurturing and teaching children under the age of six years, as well as constantly interacting with their parents [1, 2].

However, an under-considered aspect of preschool teaching profession is that the job requires the mobilization not only of emotional, but also of physical resources. Teachers are constantly required to lift, bend, or carry children as well as sit on small furniture or on the floor to take care of, play with, and interact with children [3]. Wortman [4] described teachers’ tasks as requiring constant interaction with active (sometimes hyperactive), spontaneous, impulsive, heavy (sometimes very heavy) children. Previous studies have clearly demonstrated how these kinds of physical demands negatively impact on preschool teacher musculoskeletal system [5–8]. According to the literature review carried out by Erick and Smith [9], the incidence rate of musculoskeletal disorders (MSDs) among such workers is quite high, varying from 39 to 95%. MSDs involve various body regions, particularly the back, neck, and shoulders.

MSDs may involve a wide range of inflammatory and degenerative conditions affecting the muscles, ligaments, tendons, nerves, bones, and joints. MSDs negatively impact individual well-being, having consequences on all facets of their existence, including work life [10]. Studies carried out in many occupational contexts but rarely among preschool teachers, have focused on the practical repercussions of MSDs, highlighting the loss for individuals and organizations in terms of absenteeism [11], presenteeism [12], work inability [13], and compensation claims [14]. Previous studies have also ascertained the role of MSDs in the exacerbation of perceived physical demands [15–17]. However, a clear gap of the current literature is the little attention paid to understand whether suffering from MSDs may affect the perception of other types of demands, e.g., relational demands. Different from the physical component of the job, which definitely represent a risk factor among preschool teachers, the relational nature can be seen as a double-edged sword. On one hand, the relational nature of the job may be perceived as demanding: Kelly and Berthelsen [18] showed in a qualitative study that preschool teachers felt stressed due to their high expectations about meeting the needs of the children and their parents. On the other hand, this aspect of the job may be perceived as meaningful and stimulating: Li Grining et al. [19] found that emotional demands did not necessarily negatively affect preschool teachers’ ability to implement particular management strategies. In this view, the identification of the conditions in which, among preschool teachers, this “core demand” represents a risk factor is of importance because this knowledge may help to develop preventive measures aimed at supporting workers’ health and well-being and hence maintaining a high standard of service quality.

A further gap in the literature is that whereas it was largely demonstrated that job demands may be associated with negative consequences both for workers and organizations [20], few studies have tried to understand, either among preschool teachers or among other working populations, whether and how poor health conditions, such as in the presence of MSDs, may affect this mechanism. Moreover, the literature to date has mostly focused on the buffering factors of job demands on workers’ well-being, identifying the moderating role of some job and personal resources [20], but mostly neglecting to look for the “exacerbators” of job demands. The present study may contribute to advance the literature as it is aimed to identify the psychological processes that suffering from MSDs may set in motion in workers, examining whether and how it may lead to a change in workers’ attitudes toward their job.

In particular, using the conservation of resources theory (COR) [21] as a theoretical framework, we propose that suffering from MSDs represents a condition in which relational demands may turn into a risk factor, being associated with negative attitudes toward work, i.e., perceiving their work as less meaningful and the desire to leave their job. Equally, we propose that, among preschool teachers free from MSDs, relational demands do not represent a risk factor, not showing associations with both meaning of work and intention to leave.

Work meaning refers to the degree to which the work is perceived as meaningful, important, and constructive [22]. In COR terms, work meaning may be defined as a positive motivational-affective state of fulfillment that may enhance the energetic process and thus favor the gaining of further resources. On the other hand, COR theory suggests that intention to leave represents a withdrawal strategy that individuals may plan to put into action to interrupt the loss spiral experienced [23].

COR [21] is a motivational theory based on the idea that individuals strive to obtain, retain, foster, and protect resources. Primary resources are those related to basic and survival aspects. Examples are health, shelter, and basic social needs [21]. People may instinctively seek such primary resources. Secondary resources are culturally defined and may aid people in gaining or protecting their primary resources (e.g., social status, money, and professional identity). A basic tenet of this theory is the concept of the loss spiral*,* which refers to the process of the depletion of resources that makes people unable to cope with future loss, thus potentially leading to further resource loss. Conversely, a strong armamentarium of resources tends to generate other resources, thus creating resource caravans, which may result in positive outcomes such as greater well-being and less job-related strain symptoms [24].

In personal as well as in professional life, a key aspect that considerably affects these processes is an individual’s intrinsic energetic state. According to Hobfoll and Shirom [25], the energetic state helps the body to stay in line with the activities people are undertaking to effectively respond to demands they face. At work, an excellent health status can be considered a resource functioning to keep the energetic process activated. On the other hand, the emergence of MSDs may be accompanied by an alteration of the energetic state, impairing workers’ ability to adapt to their work environment. According to the COR theory [21], individuals have a limited amount/pool of personal resources (including physical/emotional/cognitive) and dealing with MSDs could drain them, leaving worker fewer resources to cope with relational demands. For instance, in the case of preschool teachers, MSDs may seriously impair their interaction with children. This may occur because the mobilization of additional effort to compensate for workers’ limited ability due to MSDs may contribute to the acumination of pain and fatigue.

Moreover, COR theory [21] states that a condition of depleted resources may be associated with secondary costs. For example, overwhelming relational demands may lead workers to raise doubts about the usefulness and meaningfulness of the job carried out and, in turn, to foster their desire to leave the job. This mechanism may be favored by the perception of a mismatch from what teachers believe users expect and what they can actually offer [26].

From an empirical point of view, studies have provided evidence for the significance of the relationships among job demands, work meaning, and intention to leave [27, 28]. Moreover, previous literature suggests the plausibility of the mediating role of work meaning between relational demands and intention to leave [29, 30]. However, no study argued that these relationships may vary as a function of the health status of the musculoskeletal system.

Therefore, based on the COR theory (Hobfoll, 1989, 21), the present paper proposes that the health status of the musculoskeletal system represents a crucial variable among preschool teachers that differentiates those who experience a loss spiral from those who do not. In particular, we identified two subgroups, based on the presence of the MSDs, i.e., “MSDs free” and “diagnosed MSDs,” and building on what was explained above, we expect that the significance of the relationship among variables under study vary as a function of the presence of the MSDs. In particular, we expected that in the group free from MSDs, relational demands, work meaning and the intention to leave will not be significantly associated each other; on the other hand, in the group reporting MSDs, we expected that work meaning will mediate the relationship between relational demands and intention to leave.

## Method

### Data collection

Data were collected by a survey conducted in 2013 and involved all the preschool teachers employed in the Educational Municipal Public Services of Turin (Italy). A self-report pencil-and-paper questionnaire was administered individually to the teachers. The data collection took place during work hours in sessions expressly organized in each school, in which a researcher from the Department of Psychology of the University of Turin was available to assist teachers in the questionnaire completion. The voluntary nature of participation and the anonymity of the data processing were ensured.

In accordance with the legal requirements of the study country (Italy), no additional ethical approval was required because no patients were involved. Moreover, no treatment, including medical, invasive diagnostics, or procedures causing psychological or social discomfort, was administered to the participants. The research also conforms to the requirements of the 1995 Declaration of Helsinki (as revised in Edinburgh in 2000).

Eight-hundred and 84 questionnaires were distributed, 776 were returned to the research group, and 677 were correctly filled out. Male teachers were excluded because of their small number (*n* = 7).

### Measures

The self-reported questionnaire included two sections. The first was dedicated to collecting socio-demographic data (age and gender).The second section included scales aimed at measuring relational demands, work meaning, and the intention to leave as well as MSDs measures. As explained below, all the scales employed in the present study were already used or validated in previous study.

Relational demands (RDs) were measured with five items referring to relationships with children and families (e.g., “the demands of our recipients are exorbitant”, m = 2.82, sd = .67) from the Customer-Related Social Stressors (CSS) inventory developed by Dormann and Zapf [31] and adapted for the preschool context in a previous study [32]. Responses were given on a four-point scale ranging from 1 (“strongly disagree”) to 4 (“strongly agree”).

Work meaning was measured with the scale from the Copenhagen Psychosocial Questionnaire by Kristensen et al. [22]^1^. It consisted of three items (e.g., “Is your work meaningful?,” m = 3.58, sd = .42) and responses to all sub-scales were given on a four-point scale ranging from 1 (“strongly disagree”) to 4 (“strongly agree”).

Intention to leave the job was measured by three item by Cammann et al. [33] aimed at assessing the employee desire to quit the job (e.g., “I often think about quitting”, m = 1.71, sd = .61). Responses were given on five-point Likert type scales ranging from 1 “strongly disagree” to 4 “strongly agree.”

MSD symptoms were assessed using a section from the Nordic Musculoskeletal Questionnaire [34] which included nine forced-choice items (yes/no) identifying areas of the body causing musculoskeletal problems focusing on the following sites of the body: the neck, shoulders, upper back, elbows, low back, wrist/hands, hips/thighs, knees, and ankles/feet. Respondents were asked if they had musculoskeletal pain in any of these sites in the previous 12 months, lasting at least four days in a week (including the current week).

The section regarding MSDs also included questions aimed at collecting the following information: having received a medical diagnosis to which any of the symptoms suffered were attributed (yes/no); having consulted with a specialist (e.g., a physician, a physical therapist) in the last 12 months because of musculoskeletal pain (yes/no); having used drugs because of musculoskeletal symptoms (yes/no) in the past 12 months.

### Statistical analyses

Analyses were performed using SPSS 21 and MPLUS 8.

For the purpose of the present study, respondents were included in the study dataset only if eligible to be incorporated in one of the following two categories: “MSDs free” and “MSDs diagnosed.” A respondent was classified as “MSDs free” if she had never experienced musculoskeletal pain in the last 12 months (including the week in which the questionnaire was administered) and no medical diagnosis (e.g., back pain, hernias, arthrosis) was reported.

On the other hand, a respondent was included in the group “diagnosed MSDs” if both the following conditions were satisfied: (a) having experienced in the last 12 months, musculoskeletal pain in at least one site (neck, shoulders, upper back, elbows, low back, wrist/hands, hips/thighs, knees, and ankles/feet), for at least four days in a week; (b) having reported a medical diagnosis (e.g., back pain, hernias, arthrosis) to which any of the symptoms suffered were attributed.

Those who did not fall into any of these two categories were excluded from the present study. The choice to exclude from the subgroup “diagnosed MSDs” people not reporting a diagnosis, despite reporting musculoskeletal pains (measured with the Nordic questionnaire, [34]), was made in order to minimize the risk related to have used a self-report method in collecting MSDs [35], which may consist in overestimating or underestimating impairments and diseases.

As the percentage rate of missing values on each study variable was very low (less than 10%), missing values were handled by a listwise deletion procedure (i.e., an entire record is excluded from analysis if any single value is missing).

To ascertain the adequacy of the psychometric properties and the reliability of the research instruments, Cronbach’s alpha and confirmatory factor analyses (CFAs) were performed. Cronbach’s alpha was used to determine the internal consistency of each scale. On the other hand, to determine that the best structure for the data was the one hypothesized (i.e., no other latent structures underlie the data) and that measures used did not overlap each other, three models were compared with a series of CFA. The first model consisted of a one-factor model in which all items loaded on one factor. The second was a two-factor model in which the items of relational demands loaded on one factor and the rest of the items on the other factor. Finally, in the three-factor model (hypothesized factor structure), items from relational demands and work meaning and the intention to leave loaded on their corresponding factors. Because no serious violations of the normal distribution were found (all the skewness and kurtosis values of the variables considered were within ±1), maximum likelihood (ML) was employed as an estimation method. The fit of the model was assessed with the ratio of χ^2^ to the degrees of freedom (df), the comparative fit index (CFI), the Tucker-Lewis index (TLI), the standardized root mean square residual (SRMR), and the root mean square error of approximation (RMSEA). According to Kline [36], a χ^2^/df ratio of 3 or less indicates a good model fit and less than 2 indicates an excellent model fit. For TLI and CFI indices, values higher than .90 are considered indicators for good model fit [37, 38]. A value of the SRMR equal to or less than .09 indicates good fit [39]. Finally, a RMSEA value lower than .08 indicates acceptable model fit [40].

To test whether the strength of the relationships among the variables under study (i.e., relational demands, work meaning, and the intention to leave) vary as a function of MSDs, multi-group structural equation modeling (SEM) was conducted using ML. Age was included in the model as a control variable and regressed on the three major study variables [41]. The fit of the model was assessed with the ratio of χ2/df, the CFI, the TLI, the SRMR, and the RMSEA.

The mediating effects of work meaning on the relationships between relational demands and the intention to leave was assessed through both the Baron and Kenny’s method [42] and the bootstrapping procedure [43]. According to Baron and Kenny [42], the significance (*p* ≤ .05) of the following relationships suggest the presence of a mediation: (a) between the independent variable (X, i.e., relational demands) and the dependent variable (Y, i.e., intention to leave), (b) between the independent variable (X, i.e., relational demands) and the mediator (M, i.e., work meaning), and (c) between the mediator and the dependent variable (M and X, i.e., work meaning and intention to leave).

However, because several recent statistical simulation studies showed that the ability to detect mediated effects using the Baron and Kenny [42] method can be very low [44], the bootstrapping procedure was employed in order to calculate the indirect effect of relational demands on intention to leave through work meaning. In particular, the bootstrapping procedure extracted, from the original sample, 1000 bootstrap samples of the same size as the original one and estimate the indirect effect (IE) and its confidence interval (CI). A significant *p* value associated to the indirect effect and CI (95%) not including zero were both considered proves for significant mediation.

## Results

### Descriptive statistics

According to the criteria identified above, 191 preschool teachers were assigned to the category “MSDs free” and 238 to the category “diagnosed MSDs.” On the other hand, 241 were excluded by the present study.

Whereas in the MSDs-free group (*n* = 191), the average age was 47.24 (sd = 8.87; min = 29, max = 67), in the group with MSDs, the average age was 52.10 (sd = 6.35; min = 31, max = 62). ANOVA revealed significant differences among two groups on age (F = 40.91, *p* = .001).

In the group with MSDs, 84% reported pain in the upper back, 75.6% reported pain in the neck, 70.6% in the low back, 56.3% in the shoulders, 49.6% in the hips/thighs, 49.6% in the knees, 38.7% in the wrist/hands, 16.8% in the ankles/feet, and 16% in the elbows. In this group, 6.3% reported pain at only one site, 10.9% at two sites, 14.3% at three sites, 22.3% at four sites, 19.3% at five sites, 12.6% at six sites, and 14.5% at seven sites or more. Finally, 32.8% reported the regular use of drugs because of MSDs, 76.5% had consulted with a specialist (e.g., a physician, a physical therapist) in the last 12 months because of musculoskeletal pain, and 100% had received a medical diagnosis to which they attributed some or all the symptoms suffered.

### The measurement model: Multi-group CFA

Table 1 shows the goodness-of-fit indices of the three alternative measurement models estimated trough multi-group CFA on the two subgroups involved in the present study. The hypothesized model (M3) was the only one that showed an acceptable fit to the data. Moreover, Δχ^2^ tests confirmed that this three-factor model fits the data significantly better, if compared with the alternative models with two and one factors, respectively. All items in the three-factor model loaded significantly on their corresponding latent factors in the two sub-samples. In the subgroup reporting MSDs, all correlations between factors were significant (φ_relational demands-work meaning_ = −.18, *p* = .02; φ_relational demands-intention to leave_ = .39, *p* = .001; φ_work meaning -intention to leave_ = −.26, p = .001). In the subgroup free from MSDs, factors did not significantly correlate with each other (φ_relational demands-work meaning_ = .11, *p* = .22; φ_relational demands-intention to leave_ = .05, *p* = .59; φ_work meaning -intention to leave_ = −.08, *p* = .41).

**Table 1:** Multi-sample (MSDs free vs. diagnosed MSDs) confirmatory factor analyses (CFAs) – Test of alternative models – Goodness-of-fit indexes

Model	χ^2^(df)	χ^2^/df	CFI	TLI	SRMR	RMSEA	Model comparisons	Δχ^2^(p)
M1 (1 factor)	892.73 (108)	8.26	.50	.49	.13	.18 [.17–.19]	–	–
M2 (2 factors)	538.37 (104)	5.17	.72	.70	.13	.12 [.14–.15]	M2-M1	534.36 (.0001)
M3 (3 factors)	140.10 (98)	2.02	.94	.93	.06	.07 [.05–.08]	M3-M2	398.27 (.0001)

Note: *df* degree of freedom, *CFI* comparative fit index, *TLI* tucker-lewis index, *SRMR* standardized root mean square residual, *RMSEA* root mean square error of approximation

Cronbach’s alpha was found to be satisfactory since values of the scales in each group were always above .70.

### Multi-group SEM

The multi-group SEM performed across the two subgroups (MSDs Free N_listwise_ = 182, MSDs diagnosed N_listwise_ = 221) showed an acceptable fit: χ^2^ = 229.49, df = 114, CFI = .92, TLI = .91, SRMR = .07, RMSEA = .07 [.06–.08].

**Fig. 1:**
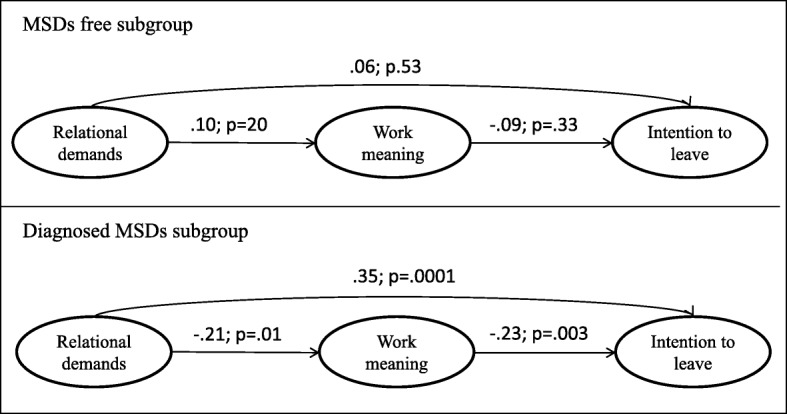
Multi-sample structural equation models across (MSDs free vs. diagnosed MSDs)

As showed in Fig. 1, in the subgroup MSDs free, nor the path from relational demands to work meaning (β = .10 *p* = .20) or the path from work meaning to intention to leave (β= − .09, *p* = .33) or the path from relational demands to intention to leave (β = .06, *p* = .53) showed significant values. In addition, age was found not to significantly affect any of the major study variables (γ_age-relational demands_ = .07 *p* = .39; γ_age-work meaning_ = .14 *p* = .08; γ_age-intention to leave_ = .07 *p* = .41).

In the subgroup with MSDs, the path from relational demands to work meaning (β= − .21, *p* = .01), the path from work meaning to intention to leave (β= − .23, *p* = .003) and the path from relational demands to intention to leave (β = .35, *p* = .0001) were all significant. In this subgroup age significantly predicted relational demands (β = .17, *p* = .02), intention to leave (β = .19, p = .02), and not work meaning (γ=.04, *p* = .54).

Moreover, in the subgroup reporting MSDs, the bootstrap test indicated the presence of a significant indirect effect of relational demands on intention to leave via work meaning in the subgroup (Standardized IE = .05, *p* = .04; CI(95%) = .01–.08).

## Discussion

Based on the COR theory [21], the aim of the present study was to test whether MSDs exacerbate the role of relational demands in lessening work meaning and, in turn, in determining intention to leave in a sample of preschool teachers. To accomplish this, from a dataset including 670 female preschool teachers employed in the educational system of the Municipality of Turin (Italy), two subgroups were selected based on the presence of the MSDs (i.e., MSDs free = 191 and diagnosed MSDs =238) and the patterns of relationships among the variables under study were examined and compared across these groups.

Overall, findings obtained confirms our expectations. In the subgroup not reporting MSDs, relational demands, work meaning, and intention to leave were found not to be significantly associated. On the other hand, in the subgroup reporting MSDs, work meaning mediated the relationship between relational demands and intention to leave.

Generally speaking, the results confirmed what was postulated based on the COR theory [21], i.e., health may be considered a crucial resource capable of deeply affecting workers’ relationship with their job. In particular, the findings suggest that physical well-being helps workers to keep available psychological resources to accomplish relational-type job demands. Therefore, our findings are consistent with the WHO perspective that conceptualizes health as a capital in which it is important to invest to achieve positive future outcomes [45].

Moreover, the present study has brought to light that the role of relational demands in affecting worker attitudes may change as a function of health conditions. In particular, for the group reporting MSDs, relational demands were found to operate as an initiator of a loss spiral, leading to diminished work meaning, and in turn, to intention to leave. On the other hand, in the subgroup free from MSDs, the loss spiral was not observed, since no significant relationships were found among the variables under study.

Therefore, our findings contain novelty, highlighting that relational demands do not inevitably represent a risk factor. Moreover, they also identified an exacerbating condition (i.e., diagnosed MSDs), never highlighted before, for relational demands. Future studies may be also directed at verifying whether the results found in the present sample, consisted of female preschool teachers, could be applied also to men. Further, to provide evidence for the generalizability of our findings, future studies should investigate whether the same mechanism is also effective in other working populations that have in common with preschool teachers the exposure to relational demands as well as to the risk to develop MSDs (e.g., nurses).

The present study has some limitations. The most relevant is its cross-sectional design. Future researchers should employ a longitudinal design to explore the cross-lagged associations between the constructs examined across various health conditions. Indeed, as suggested by the COR theory [21, 25], the relationship between all these constructs, more than unidirectional, may be cyclic. Longitudinal studies may also be useful to understand whether and how the relationship between these constructs changes over time.

Another limitation is that all of the measures employed were self-reported. Data coming from a single source may introduce the issue of common method variance [46]. Future studies may benefit from the employment of research designs that include a combination of objective and subjective measures or use data from multiple sources (e.g., the inclusion of a medical assessment for the measurement of MSDs would increase the reliability of its measure).

Finally, the representativeness of the results may be a limiting factor. The present study was conducted among a specific professional group, i.e., preschool teachers. Therefore, caution should be exercised when generalizing the results to other working populations employed as well as to other kinds of teachers.

## Conclusions

The relevance of the present paper was to demonstrate that suffering from MSDs impairs workers’ ability to invest psychological resources at work, thus activating a spiral that may encompass loss not only for such individuals, but also for the recipients of their services and their employer organizations.

From a practical point of view, the present findings suggest that organizations may take advantage of developing actions aimed at protecting and, most of all, promoting physical health. Investing in preventive measures focused on promoting physical health may be ever more important in light of the consideration that one of the most important emerging work-related issues is the aging workforce [47]. In our study, it was found that the incidence of MSDs tends to increase with age (M_MSDs free_ = 47, M_MSDs diagnosed_ = 52.10, F = 40.91 *p* = .0001). In this view, investing in workers’ physical health may represent a measure that contributes to maximizing the likelihood that workers *play as a full resource* (being healthier and more committed in their work) for a longer time during their work life span.

Finally, regarding the preschool context in particular, the present study highlighted that it is necessary to urgently intervene: a relevant segment of the sample reported MSDs and continued working despite the fact that this condition led to the development of the desire to leave their job. In this context, organizations need to develop a set of composite measures aimed to both minimize the risk to develop MSDs among those who have not yet reported them and to contain further secondary losses among who have yet to develop MSDs. First, it might be a crucial health promotion action to re-design kindergarten school facilities to convert them into an ergonomically appropriate environment to minimize the occurrence of movements that place strain on the preschool teachers’ bodies and that have potential to cause injuries to the musculoskeletal system. Second, continuing education programs should be implemented, especially if focused on learning techniques that are useful to perform daily work activities without negatively impacting the musculoskeletal system (e.g., how to correctly lift or to prevent injuries due to the assumption of awkward postures). Posture exercise classes aimed at lessening and preventing musculoskeletal pain and diseases may also be beneficial. Health promotion programs may also include informative actions aimed at enabling workers to increase control over their health and at encouraging them to adopt a healthier lifestyle (e.g., diet modification, the introduction of physical activities). Finally, but no less important, it seems necessary to identify specific interventions for those who report MSDs that should be tailored to the specific needs/characteristics of the workers. This may include rehabilitation treatment and, in the case in which MSDs is serious or not reversible, it should be combined with programs that accompany workers to job replacement (e.g., for younger) or early retirement (e.g., for older that have not yet reached the retirement age).

However, considering the relevance of MSDs issue among preschool teachers, in addition to the engagement of the single organization in investing in preventive/protective measures, the development of specific guidelines and recommendations from the international health and safety public agencies may be necessary to help employer organizations and country governments to correctly identify measures to address the problem of MSDs among preschool teachers.

## Abbreviations

ANOVA: analysis of variance
CFA: confirmatory factor analysis
CFI: comparative fit index
COR: conservation of resource
df: degrees of freedom
ML: maximum likelihood
MSDs: musculoskeletal disorders
RMSEA: root mean square error of approximation
SEM: structural equation modeling
SRMR: standardized root mean square residual
TLI: Tucker-Lewis index

## Footnotes

1 Items of this subscales were reported in the following document: http://www.arbejdsmiljoforskning.dk/~/media/Spoergeskemaer/copsoq/english-copsoq-2-ed-2003-pdf.pdf. Last access: 4th May, 2018.
